# Electrophysiological characteristics of paraventricular thalamic (PVT) neurons in response to cocaine and cocaine- and amphetamine-regulated transcript (CART)

**DOI:** 10.3389/fnbeh.2014.00280

**Published:** 2014-08-22

**Authors:** Jiann Wei Yeoh, Morgan H. James, Brett A. Graham, Christopher V. Dayas

**Affiliations:** Neurobiology of Addiction Laboratory, School of Biomedical Sciences and Pharmacy, and The Centre for Translational Neuroscience and Mental Health Research, University of Newcastle and the Hunter Medical Research InstituteNewcastle, NSW, Australia

**Keywords:** CART, addiction, cocaine, reward-seeking, midline thalamus, electrophysiology, PVT

## Abstract

Recent work has established that the paraventricular thalamus (PVT) is a central node in the brain reward-seeking pathway. This role is mediated in part through projections from hypothalamic peptide transmitter systems such as *cocaine- and amphetamine-regulated transcript (CART)*. Consistent with this proposition, we previously found that inactivation of the PVT or infusions of CART into the PVT suppressed drug-seeking behavior in an animal model of contingent cocaine self-administration. Despite this work, few studies have assessed how the basic physiological properties of PVT neurons are influenced by exposure to drugs such as cocaine. Further, our previous work did not assess how infusions of CART, which we found to decrease cocaine-seeking, altered the activity of PVT neurons. In the current study we address these issues by recording from anterior PVT (aPVT) neurons in acutely prepared brain slices from cocaine-treated (15 mg/ml, *n* = 8) and saline-treated (control) animals (*n* = 8). The excitability of aPVT neurons was assessed by injecting a series of depolarizing and hyperpolarizing current steps and characterizing the resulting action potential (AP) discharge properties. This analysis indicated that the majority of aPVT neurons exhibit tonic firing (TF), and initial bursting (IB) consistent with previous studies. However, we also identified PVT neurons that exhibited delayed firing (DF), single spiking (SS) and reluctant firing (RF) patterns. Interestingly, cocaine exposure significantly increased the proportion of aPVT neurons that exhibited TF. We then investigated the effects of CART on excitatory synaptic inputs to aPVT neurons. Application of CART significantly suppressed excitatory synaptic drive to PVT neurons in both cocaine-treated and control recordings. This finding is consistent with our previous behavioral data, which showed that CART signaling in the PVT negatively regulates drug-seeking behavior. Together, these studies suggest that cocaine exposure shifts aPVT neurons to a more excitable state (TF). We propose that the capacity of CART to reduce excitatory drive to this population balances the enhanced aPVT excitability to restore the net output of this region in the reward-seeking pathway. This is in line with previous anatomical evidence that the PVT can integrate reward-relevant information and provides a putative mechanism through which drugs of abuse can dysregulate this system in addiction.

## Introduction

It has been almost 10 years since the publication of the highly influential proposal of a hypothalamic-thalamic-striatal axis that is critical to the regulation of energy balance, arousal, and food reward (Kelley et al., 2005). Central to this model was a role for the paraventricular thalamus (PVT) as a key interface between energy and reward-related hypothalamic signaling and major output pathways, including the striatum and cortex. The PVT is unique among the midline and intralaminar thalamic nuclei in that it receives significant projections from several areas of the hypothalamus, including the arcuate, dorsomedial, ventromedial and lateral hypothalamic nuclei. These inputs provide a key source of neuropeptides including *cocaine- and amphetamine-regulated transcript* (CART; Kirouac et al., 2006; Parsons et al., 2006; Li and Kirouac, 2012), which was originally identified through its roles in feeding behavior (Kristensen et al., 1998; Sakurai et al., 1998). Findings that individual PVT neurons receive inputs from both “pro” and “anti”-reward peptides has further fueled interest in this brain region as a key modulator of motivation and reward (Deutch et al., 1998; Hamlin et al., 2009; James et al., 2010, 2011a,b; Martin-Fardon and Boutrel, 2012).

Importantly, a series of recent studies have demonstrated that the PVT modulates expression of drug-seeking behavior. For example, drug-related cues recruit neurons in the PVT (Brown et al., 1992; Franklin and Druhan, 2000; Wedzony et al., 2003; Dayas et al., 2008; James et al., 2011b), and inactivation of the PVT suppresses both alcohol- (Hamlin et al., 2009) and cocaine- (James et al., 2010) seeking behavior. Based upon Kelley’s (2005) original proposal, we and others have explored the role of PVT in acting as a relay of drug-relevant hypothalamic neuropeptide activity to brain regions such as the nucleus accumbens (NAC) or prefrontal cortex (PFC; Hamlin et al., 2009; James et al., 2012; Martin-Fardon and Boutrel, 2012). To date, relatively little is known about the effects of drug exposure on PVT neurons or the effects of neuromodulators such as CART on the excitatory drive to this population.

The highest density of CART producing neurons is found within arcuate and lateral hypothalamic nuclei (Elias et al., 2001; Vrang, 2006). Early studies demonstrated that CART negatively regulates feeding behavior to promote satiety (Kristensen et al., 1998; Lambert et al., 1998) and the available evidence suggests that CART similarly suppresses drug-motivated behaviors (Jaworski et al., 2003; Dayas et al., 2008; Hubert et al., 2010; King et al., 2010; Yoon et al., 2010). For example, intra-PVT infusions of the CART 55–102 peptide attenuated reinstatement of cocaine-seeking (James et al., 2010). These findings suggest that CART might suppress the activity of PVT neurons and thereby block addiction-related reward pathway signaling, however, this hypothesis remains to be directly tested.

The primary aim of the present study is to investigate the cocaine-induced changes in PVT neuron excitability. To do this, we made patch-clamp recordings in brain slices from mice exposed to repeated systemic injections of cocaine and examined the intrinsic excitability of PVT neurons. We also investigated the effect of CART on excitatory synaptic input to PVT neurons in both cocaine-exposed and control animals.

## Materials and methods

### Animals

All experimental procedures were approved by the University of Newcastle Animal Care and Ethics Committee and performed in accordance with the New South Wales Animal Research Act, Australia. Male C57BL/6 mice (*n* = 16, The University of Newcastle, NSW, Australia) aged 4–8 weeks were housed four per cage in temperature- and humidity-controlled conditions on a normal daylight cycle (lights on at 0700 and off at 1900) with *ad libitum* access to food and water. All experiments were carried out during the animals’ dark active phase.

### Drugs

Cocaine Hydrochloride (GlaxoSmithKline, Victoria, Australia) was dissolved in sterile physiological saline: (15 mg/ml) for intraperitoneal injection. Drugs for electrophysiology were made at 1000 times stock concentrations and then diluted to the final concentration in bath superfusate. Picrotoxin was purchased from Sigma-Aldrich (St. Louis, MO, USA) and CART was purchased from Tocris Bioscience (Bristol, UK).

### Cocaine exposure procedures

Prior to treatment, animals were conditioned to daily handling (1 h/day for 3 days) and were subjected to single-daily sham injections, after which they were placed in an enclosed arena (50 cm × 50 cm) for 1 h. Animals were then randomly allocated into two treatment groups; one group receiving saline injections (vehicle, *n* = 8) and the other group received cocaine injections (15 mg/kg; i.p., *n* = 8) for seven consecutive days (Yeoh et al., 2012). Immediately following injections, animals were placed in the enclosed arena for 1 h before they were returned to their home cage. Brain slice electrophysiology experiments were undertaken 24 h after the last cocaine/saline injection session during the dark phase.

### Slice preparation for electrophysiology experiments

Mice were deeply anesthesized with ketamine (100 mg kg^−1^ i.p) and decapitated. Brains were rapidly removed and immersed in ice-cold oxygenated (95% O_2_, 5% CO_2_) sucrose substituted artificial cerebrospinal fluid (S-ACSF, containing in mM: 236.2 sucrose, 25 NaHCO_3_, 13.6 glucose, 2.5 KCl, 2.5 CaCl_2_, 1 NaH_2_PO_4_ and 1 MgCl_2_) maintained at 4°C by a temperature-controlled tissue-slicing bath (Model 7610, Campden, Leicester, England). Coronal slices (250 μm) containing anterior PVT (aPVT) were obtained using a vibrating microtome (Campden 7000 smz, Leicester, England), then transferred to an oxygenated storage chamber containing artificial cerebrospinal fluid (ACSF—119.4 mM NaCl substituted for sucrose) and incubated for a minimum of 1 h at room temperature prior to recording.

### Electrophysiology

Slices were transferred to a recording chamber and continually superfused with oxygenated ACSF, maintained at ~33°C by an inline temperature controller (TC324B, Warner Instruments, Hamden, USA). Neurons were visualized using infrared differential interference contrast microscopy. Whole-cell recordings were made using a Multiclamp 700B amplifier (Molecular Devices, Sunnyvale, CA), digitized at 10 kHz, via an ITC-18 computer interface (Instrutech, Long Island, NY) and recorded onto a Macintosh computer running Axograph X software (Axograph). All recordings were restricted to the aPVT region, spanning between Bregma −0.46 and −0.94 (Paxinos and Franklin, 2001). Recording pipettes (4–6 MΩ) were filled with an internal solution containing (in mM): 135 KCH_3_SO_4_, 6 NaCl, 2 MgCl_2_, 10 HEPES, 0.1 EGTA, 2 MgATP, 0.3 NaGTP (pH 7.3 with KOH). After obtaining the whole-cell recording configuration, series resistance and input resistance were calculated based on the response to a −5 mV voltage step from a holding potential of −70 mV. These values were monitored at the beginning and end of each recording and data were rejected if values changed by more than 20%. Spontaneous excitatory postsynaptic currents (sEPSCs) were recorded in voltage clamp at a holding potential of −70 mV with series resistance of <25 MΩ, in the presence of picrotoxin (100 μM). In some recordings, 10 nmol CART was bath applied to slices for a minimum of 3 min before the data was collected for sEPSCs analysis (Davidowa et al., 2003). All data were corrected for liquid junction of −14 mV due to our potassium methyl sulphate internal.

Action potential (AP) discharge was studied in current-clamp mode. The membrane potential recorded 30 s after switching from the voltage-clamp to the current clamp was designated as the resting membrane potentials (RMP). AP discharge was studied by injecting a series of depolarizing current-steps (20 pA increments, 800 ms duration) from RMP, delivered every 5 s. During this protocol, voltage deflections were limited to avoid cell damage with the protocol terminated if the sustained depolarization (i.e., outside periods of AP discharge) exceeded −20 mV or the neurons failed to elicit AP.

### Data analysis

Data analysis was performed offline using semi-automated procedures within Axograph X (v1.3.5). A two-template method was used to capture the sEPCSs (Clements and Bekkers, 1997). sEPSCs were first detected and captured using a sliding template, along with a minimum amplitude threshold criteria of 10 pA. Captured sEPSCs were individually inspected and excluded if they had an unstable baseline before the rise or during the decay phase of the sEPSC, or contained overlapping sEPSCs. All accepted sEPSCs were then averaged and the resulting trace was used as a final template for detection of sEPCSs to be used for analysis. As the second template is generated using the sEPSCs of PVT neurons, the semi-automated sliding template procedure within Axograph allowed us to efficiently detect small currents near the background noise level. Peak amplitude, rise-time (calculated over 10–90% of peak amplitude) and decay time constant (calculated over 20–80% of the decay phase) were obtained. Average sEPSC frequency was calculated by dividing the number of captured events by the search duration in seconds. sEPSCs were compared across saline- and cocaine-treated animals using individual independent samples *t*-tests. The effect of CART on sEPSCs was examined in a representative subset of recordings that retained mean sEPSC values of both saline- and cocaine treated-animals, as assessed by independent-samples *t*-tests. The effect of CART on sEPSCs properties in these recordings was examined using individual paired-sample *t*-tests. Cumulative probability distributions were generated for instantaneous frequency, calculated as the inverse of inter-event interval for successive sEPSCs.

**Figure 1:**
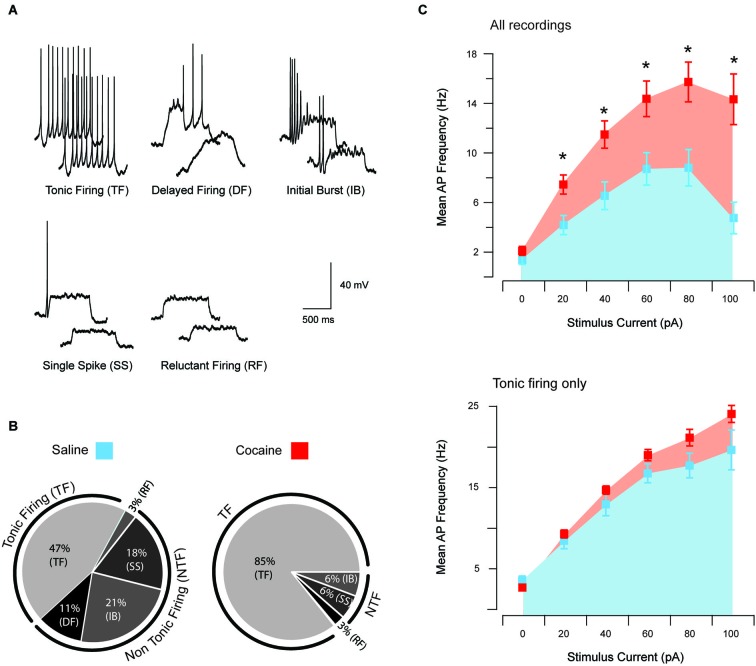
**Action potential discharge and current-discharge frequency of anterior PVT neurons**. Five distinct patterns of AP discharge were observed in aPVT neurons during response to current step injections of increasing amplitude; tonic firing (TF), delayed firing (DF), initial burst (IB), single spike (SS), and reluctant firing (RF)** (A)**. All five AP firing patterns were observed in recordings from saline-exposed animals, with 44% exhibiting TF, 11% DF, 24% IB, 18% SS and 3% RF **(B, left)**. In contrast, cocaine-exposed animals only showed four of the five described AP firing patterns, with 85% exhibiting TF, 3% RF, 6% SS and 6% IB **(B, right)**. A chi-squared analysis revealed a significant interaction between cocaine treatment and firing pattern type, *χ*^2^ (4, *n* = 73) = 13.53, *p* = 0.009. Recordings from cocaine-exposed animals reached higher mean AP discharge frequency at all current step injections above 20 pA **(C, upper)**. In contrast, the same comparison of AP discharge frequency, specifically in tonic firing PVT neurons from both treatment groups, did not differ **(C, lower)**.

Passive membrane properties were compared across treatment groups using independent samples *t*-tests. In instances where data were not normally distributed, a non parametric Kruskal-Wallis test was used. For AP analysis, rheobase current was defined as the minimum current step to elicit at least a single AP. The AP discharge was classified according to the spiking pattern observed during current injection (Graham et al., 2004). Tonic firing was characterized by persistent AP discharge throughout the depolarizing protocol; initial burst AP discharge was limited to the beginning of the depolarizing protocol; delayed firing discharge featured a delay before AP was elicited; single spike was characterized by the discharge of a single AP, irrespective of the amplitude of the current step injected; and reluctant firing (RF) was characterized as not discharging AP (Figure 1). Importantly, this classification was applied using data collected at RMP in order to capture the preferred AP discharge exhibited by neurons under saline and cocaine-treated conditions.

AP discharge patterns were compared across treatment groups using a chi-squared test. Discharge frequency during depolarizing current-steps was assessed using a 2 “treatment” (saline, cocaine) × 6 “current” (0, 20, 40, 60, 80, 100 pA) mixed model ANOVA. In the case of a significant interaction, *post hoc* *t*-tests were carried out to compare treatment groups at each current step and the alpha value was adjusted for family wise error. Mean AP discharge frequency was quantified by calculating the numbers of APs in each step responses and dividing this value by the current step duration (800 ms).

## Results

### Effect of cocaine on the intrinsic excitability of aPVT neurons

Patch clamp recordings were obtained from aPVT neurons in saline- and cocaine-exposed animals (38 vs. 35 neurons, respectively). These recordings could be coarsely separated into two groups based on whether neurons responded to a depolarizing step protocol with repetitive AP discharge (tonic firing) or not (non-tonic firing). Comparisons of series resistance, input resistance, RMP and rheobase current in tonic firing and non-tonic firing neurons recorded from saline- and cocaine-exposed animals are summarized in Table 1. Series resistance was similar across all groups, suggesting that recording conditions do not contribute to the differences described below. Interestingly, input resistance was selectively increased in cocaine tonic firing (CTF) neurons compared to all other groups. RMP was similar between CTF and saline tonic firing (STF) groups, and also between cocaine non-tonic firing (CNTF) and saline non-tonic firing (SNTF) groups. Finally, rheobase currents were significantly lower in the tonic firing groups vs. non-tonic firings groups under saline and cocaine treated conditions.

**Table 1:** **Passive membrane properties of aPVT neurons in saline- and cocaine-exposed animals**.

**Treatment**	**Saline**		**Cocaine**	
**Firing pattern**	**Tonic firing (STF)**	**Non tonic firing (SNTF)**	**Tonic firing (CTF)**	**Non tonic firing (CNTF)**
*n*	18	20	30	5
Input resistance (MΩ)	225.57 ± 17.81	199.80 ± 20.09	305.46 ± 20.66 Θ	202.14 ± 24.15
Series resistance (MΩ)	9.98 ± 0.95	8.62 ± 0.61	9.59 ± 0.69	8.44 ± 0.45
RMP (mV)	−57.16 ± 1.16	−55.69 ± 2.25	−59.11 ± 0.80 +	−60.10 ± 3.62
Rheobase current (pA)	8.89 ± 3.32 Δ	43.00 ± 11.26	6.67 ± 1.75 *ε*	88.00 ± 26.53

*Values are mean ±SEM. Significant differences (*p* < 0.05) is denoted by different symbols according to the comparison groups; Θ significant compared to STF, SNTF and CNTF, + significant compared to SNTF, Δ significant to SNTF and CNTF, ε significant to SNTF and CNTF*.

Responses to the depolarizing step protocol could be further differentiated into five categories based on multiple current injection responses (Figure 1A); tonic firing (TF), delayed firing (DF), initial burst (IB), single spiking (SS) and RF. In saline-exposed animals, TF accounted for 47% of the sample population, followed by 21% IB, 18% SS, 11% DF and 3% RF (Figure 1B). In contrast, only four AP discharge patterns were observed in cocaine-exposed animals, with TF accounting for 85% of the sample population followed by 6% of IB, 6% of SS and 3% of RF (Figure 1B). A chi-squared analysis revealed a significant interaction between cocaine exposure and firing type *χ*^2^ (4, *n* = 73) = 13.53, *p* = 0.009. Thus, cocaine exposure shifted the intrinsic excitability of aPVT neurons to a more excitable mode of AP discharge.

To further examine the shift in excitability of aPVT neurons from cocaine-exposed animals, we compared the injected current/discharge frequency relationship in both treatment groups (Figure 1C, upper). A repeated measures ANOVA analysis revealed a significant main effect of “group” (saline vs. cocaine; *F*_(1,77)_ = 14.62, *p* < 0.001), indicating that across all recordings, mean discharge frequencies were significantly greater in cocaine-exposed animals. A predictable significant main effect of “current” (0, 20, 40, 60, 80 and 100 pA) was also observed, indicating that discharge frequencies increased with increasing current injections (*F*_(5,385)_ = 43.71, *p* < 0.001), with 80 pA producing the highest discharge frequency in both treatment groups. Most importantly, we observed a significant interaction between “group” and “current” (*F*_(5,385)_ = 6.46, *p* < 0.001), indicating that the relationship between current injection and discharge frequency was different in cocaine- vs. saline-exposed animals. *Post hoc* analyses revealed that whilst treatment groups did not differ in terms of discharge frequency at their RMP (*p* = 0.13), this parameter was significantly higher in cocaine-exposed animals at 20 pA (*p* = 0.003), 40 pA (*p* = 0.002), 60 pA (*p* = 0.005), 80 pA (*p* = 0.002) and 100 pA (*p* < 0.001; adjusted alpha value for all comparisons, *p* = 0.008). Therefore, consistent with the shift to a more excitable form of AP discharge (tonic firing), we found that the input/output transformation of aPVT neurons in cocaine-exposed animals was elevated.

In addition to the above analysis we repeated our comparison of the injected current/discharge frequency relationship specifically in tonic firing neurons from both saline- and cocaine-exposed animals (Figure 1C, lower). This was undertaken to differentiate between the possibility that this altered current/discharge relationship was due to the increase in the proportion of tonic firing neurons under cocaine-exposed conditions, or a selective increase in the AP discharge frequency of tonic firing neurons. A repeated measures ANOVA revealed a significant main effect of “current”, confirming that discharge frequencies were significantly elevated at increasing current steps (*F*_(5,140)_ = 187.08, *p* < 0.001). We did not observe a main effect of “treatment” (*F*_(1,28)_ = 3.23, *p* > 0.05). No interaction was detected, however, between “current” and “treatment” (*F*_(5,140)_ = 1.14, *p* > 0.05), indicating that increases in current affected discharge frequencies in cocaine- and saline-treatment animals equally. When taken together, these results indicate that cocaine did not alter the current/firing frequency relationship of tonic firing neurons. Rather, the shift to a greater proportion of aPVT neurons exhibiting tonic firing discharge caused an overall enhancement of excitability in this region.

### Effect of cocaine on excitatory synaptic transmission of aPVT neurons

**Figure 2:**
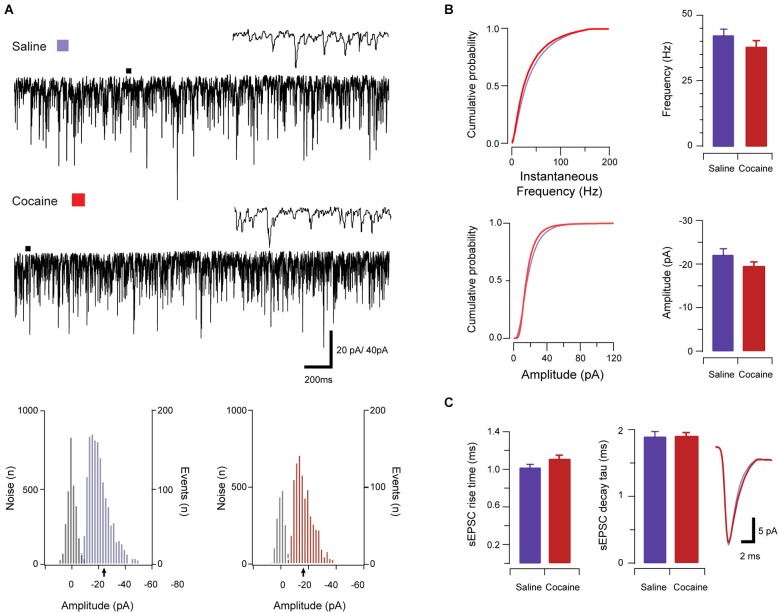
**Spontaneous excitatory synaptic transmission in saline- and cocaine-exposed animals**. Traces show 3 s of continuous recordings from aPVT neurons in saline- **(A, upper)** and cocaine-exposed animals **(A, middle)**, with corresponding amplitude histograms (including baseline noise distributions) below and arrows indicating the mean (saline, mean amplitude = −22 pA, **A, lower left**; cocaine, mean amplitude = −18 pA **A, lower right**). Cumulative probability distributions (from representative recordings) and bar graphs showed no differences in both instantaneous frequency (**B, upper**) and amplitude (**B, lower**) of aPVT neurons in response to saline or cocaine. Group data plots (**C**) show rise time and decay time constant remained similar in both saline and cocaine treatment groups (*n* = 25 and *n* = 24, with an average of 1387 events and 1192 events analyzed respectively), with representative traces of rise and decay.

A total of 49 neurons (saline: *n* = 25; cocaine: *n* = 24) were analyzed in this dataset to assess the effects of cocaine on excitatory synaptic transmission onto aPVT neurons. Given the above data showing that the intrinsic excitability of aPVT neurons is elevated by cocaine, sEPSCs were recorded in saline- and cocaine-exposed animals to assess the strength of synaptic drive to this population. sEPSCs, which include currents arising from spontaneous and AP-dependent neurotransmitter release, were recorded in the presence of 100 μM picrotoxin to block inhibitory GABA_A_ receptor-mediated currents (Figure 2A). sEPSC properties were similar across treatment groups. Specifically, sEPSC frequency (saline vs. cocaine, 42.56 Hz ± 2.39 Hz vs. 37.97 Hz ± 2.46 Hz, *p* > 0.05; Figure 2B), amplitude (saline vs. cocaine, −22.98 pA ± 1.45 pA vs. −19.40 pA ± 0.99 pA, *p* > 0.05; Figure 2B), rise time (1.02 ms ± 0.03 ms vs. 1.11 ms ± 0.04 ms, respectively; Figure 2C), and decay time constant (1.89 ms ± 0.08 ms vs. 1.90 ms ± 0.05 ms, respectively; Figure 2C), were indistinguishable between saline- and cocaine-exposed animals (*p*’s > 0.05, independent samples *t*-tests). Thus, unlike the intrinsic excitability of aPVT neurons, spontaneous excitatory synaptic transmission to this population does not appear to be affected by cocaine exposure. When combined, these results suggest that a normal level of synaptic drive synapses on a more excitable population of aPVT neurons, which would be predicted to enhance the output of this region under cocaine-exposed conditions.

### Effect of CART on excitatory synaptic transmission in saline- and cocaine-exposed animals

**Figure 3:**
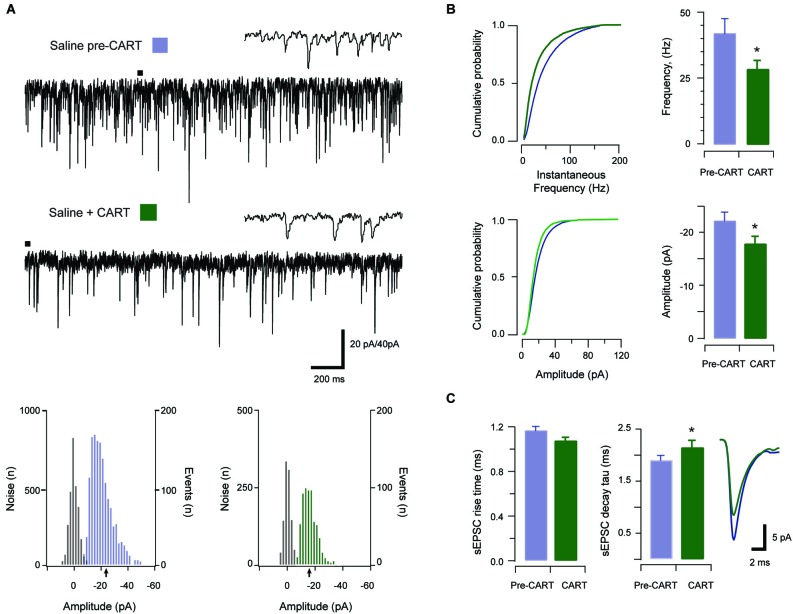
**The effect of CART on spontaneous excitatory synaptic transmission in saline-treated animals**. Traces show 3 s of continuous recording from an aPVT neuron prior to **(A, upper)**, and then during bath application of 10 nmol CART **(A, middle)**, with corresponding amplitude histograms (including baseline noise distributions) below and arrows indicating the mean (saline pre-CART, mean amplitude = −22 pA, **A, lower left**; saline post-CART, mean amplitude = −17 pA, **A, lower right**). Cumulative probability distributions (from representative recordings) showed a significant reduction in both instantaneous frequency (**B, upper**) and amplitude (**B, lower**) of aPVT neurons in response to saline or cocaine. Group data plots (**C**) show sEPSC instantaneous frequency was reduced by CART (* *p* = 0.002**)**, as was amplitude (* *p* = 0.008**)**. In contrast, rise time was not affected by CART. Finally, decay time constant was significantly increased following CART (* *p* = 0.007, an average of 1387 events and 799 events were analyzed respectively) with representative traces of rise and decay time pre- and post-CART.

**Figure 4:**
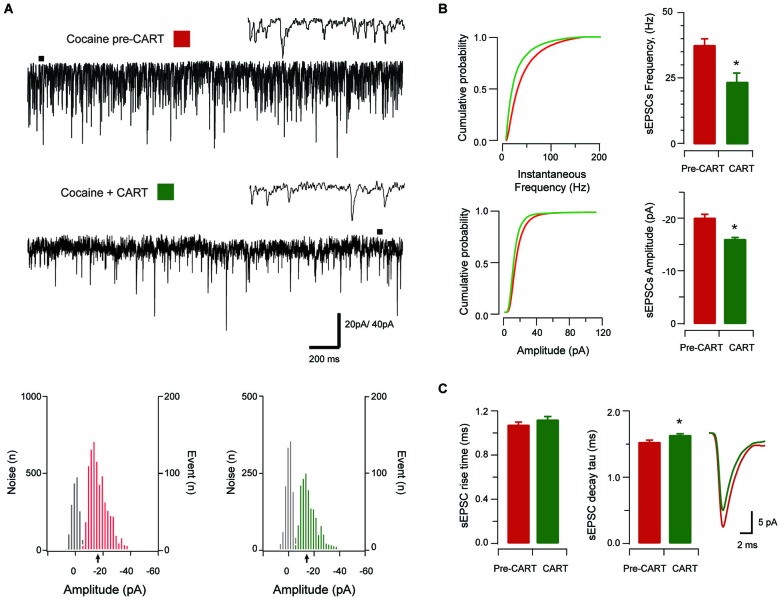
**The effect of CART on spontaneous excitatory synaptic transmission in cocaine-treated animals**. Traces show 3 s of continuous recording from an aPVT neuron prior to **(A, upper)**, and then during bath application of 10 nmol CART **(A, middle)**, with corresponding amplitude histograms (including baseline noise distributions) below and arrows indicating the mean (cocaine pre-CART, mean amplitude = −18 pA, **A, lower left**; cocaine post-CART, mean amplitude = −16 pA, **A, lower right**). Cumulative probability distributions (from representative recordings) showed a significant reduction in both instantaneous frequency (**B, upper**) and amplitude (**B, lower**) of aPVT neurons in response to saline or cocaine. Group data plots (**C**) show sEPSC instantaneous frequency (* *p* < 0.001**)**, and amplitude (* *p* < 0.001**)** were both reduced after CART application. We did not observe a significant effect of CART on rise time, however CART application resulted in a significant increase in decay time constant (* *p*** =** 0.11, an average of 1192 events and 669 events were analyzed respectively), with representative traces of rise and decay time pre- and post-CART.

Given the previous behavioral data showing that CART exerts an inhibitory effect on cocaine related behavior in the PVT, we next sought to determine if these observations might be mediated through an effect on excitatory synaptic transmission in the aPVT. CART was bath applied during a subset of sEPSC recordings from saline- and cocaine-exposed animals (saline *n* = 11 (Figure 3A) and cocaine *n* = 13 (Figure 4A) respectively). Importantly, the recordings used to study CART-effects were statistically indistinguishable from the complete sample for sEPSC frequency, amplitude, rise time and decay time constant (*p*’s > 0.05) for both treatment groups.

In saline-treated animals, sEPSCs frequency was significantly reduced by CART application (baseline vs. CART, 41.65 Hz ± 4.59 Hz vs. 28.59 Hz ± 3.32 Hz, *t*_(10)_ = 4.06, *p* = 0.002, paired samples *t*-test, Figure 3B). Likewise, sEPSC amplitude was also significantly reduced by CART (baseline vs. CART, −23.99 pA ± 2.86 pA vs. −17.87 pA ± 1.43 pA, *t*_(10)_ = −3.32, *p* = 0.008, paired-samples *t*-test, Figure 3B). In contrast, rise time (baseline vs. CART, 1.14 ms ± 0.05 ms vs. 1.07 ms ± 0.04 ms, *p* = 0.141; Figure 3C) was not affected by CART treatment. Finally, decay time constant (baseline vs. CART, 1.82 ms ± 0.11 ms vs. 2.15 ms ± 0.14 ms, Figure 3C) was significantly increased following CART application (*t*_(10)_ = −3.40, *p* = 0.007, paired samples *t*-test; Figure 3C). Thus, under control (saline-treated) conditions, CART was able to significantly reduce excitatory drive in the aPVT.

In cocaine-exposed animals, application of CART also reduced sEPSCs frequency (cocaine vs. CART, 36.89 Hz ± 2.76 Hz vs. 23.92 Hz ± 3.45 Hz, *t*_(12)_ =5.82, *p* < 0.001, paired samples *t*-test, Figure 4B), and amplitude (−20.23 pA ± 1.49 pA vs. −16.01 pA ± 0.81 pA, *t*_(12)_ =−5.12, *p* < 0.001, paired samples *t*-test, Figure 4B). Again, there was no effect of CART application on rise time (baseline vs. CART, 1.05 ms ± 0.07 ms vs. 1.10 ms ± 0.05 ms, *p* = 0.051, paired-samples *t*-test; Figure 4C), however, decay time constant was significantly increased following CART application (1.86 ms ± 0.65 ms vs. 2.03 ms ± 0.06 ms, *t*_(12)_ = −3.02, *p* = 0.011, paired-samples *t*-test; Figure 4C). Thus, CART retains its capacity to reduce excitatory drive in the aPVT of cocaine-exposed animals and may therefore be useful in returning the activity of aPVT neurons to control levels.

## Discussion

In this study, we assessed the effects of cocaine exposure on the intrinsic excitability and synaptic drive to aPVT neurons. In addition, we examined the effects of CART on excitatory synaptic transmissions in the aPVT in both cocaine- and saline-treated animals, prompted by accumulating anatomical and behavioral evidence indicating that the aPVT is a site of convergence for hypothalamic signaling. Below, this data is discussed in the context that PVT neurons regulate state-dependent arousal, a function that is highly relevant to addiction processes and likely to be modulated by hypothalamic feeding peptides including CART.

Consistent with previous studies, we show that the predominant AP discharge of PVT neurons is tonic and burst firing (Kolaj et al., 2007, 2012, 2014; Wong et al., 2013) However, in addition to these typical thalamocortical firing patterns, we also observed small proportions of neurons that exhibited DF, SS and RF. There are a number of differences between our experiments and this previous literature that may contribute to these variations. For example, our study undertook all recordings from PVT slices prepared from animals during the dark (active) phase whereas the majority of literature has assessed PVT neuron activity in slices prepared from animals during the light (inactive) phase. Recently, Kolaj et al. (2012) demonstrated that the conductance, RMP, and AP firing patterns in midline PVT neurons vary during the active vs. inactive phases. These findings suggest that the intrinsic properties of PVT neurons are state-dependent, and as such, the DF, SS and RF might be more easily identified under certain environmental conditions. It also important to highlight that the present study used C57BL/6 mice, whereas other electrophysiological studies examining thalamocortical or midline PVT neurons have used guinea pigs (Jahnsen and Llinás, 1984) or Sprague-dawley rats (Wong et al., 2013). Finally, the experimental conditions such as recording temperature and membrane potential have been shown to affect the excitability of CNS neurons (Graham et al., 2008; Buzatu, 2009). In the present study we collected our data at elevated temperatures (~33°C) whereas previous studies have been typically undertaken at room temperature, which will alter the properties of the many conductances’ that combine to shape AP discharge. In addition, our study assessed AP discharge from the RMP observed soon after recordings commenced. This approach was chosen to capture any cocaine-related effects on membrane potential, which could influence AP discharge properties. In contrast, most previous studies have injected current into PVT neurons, adjusting membrane potential to a set level to assess AP discharge. Importantly, the RMP in our experiments (−57 mV vs. −61 mV, saline- and cocaine-exposed recordings) are similar to the membrane potentials reported by other groups (−60 mV), and therefore this factor is unlikely to account for the differences seen in our recordings (Deschênes et al., 1982; Jahnsen and Llinás, 1984; Kolaj et al., 2007; Wong et al., 2013).

Regardless of the subtle differences in discharge properties between previous studies and ours, a key finding from our work was an almost twofold increase in PVT neurons displaying tonic firing in cocaine-exposed animals. This was accompanied by a concomitant reduction in PVT neurons exhibiting IB, SS, and RF discharge patterns and absence of the DF discharge pattern. Further analysis showed that the input/output relationship, assessed by mean AP discharge frequency, was enhanced in the cocaine-exposed group. This increase in AP discharge frequency was caused by a shift from other discharge patterns to tonic firing in cocaine-exposed animals, rather than an increase in the rate of AP discharge exhibited by tonic firing PVT neurons.

The conversion of other firing patterns to tonic firing might be explained by the increase in input resistance in cocaine-treated animals. This would be expected to increase the level of depolarization due to each current step injection and therefore enhance the stimulus to discharge APs. Other work has also shown that AP discharge in the PVT is highly mutable via a number of mechanisms. For example, Wong et al. (2013), recently showed that Ca^2+^ influx through N-type Ca^2+^ channels is important in the modulation of tonic firing in PVT neurons. They also demonstrated that blockade of transient receptor potential (TRP) family of ion channels enhanced AP firing while application of small-conductance Ca^2+^-dependent K^+^ (SK) antagonist reduced AP firing. It is therefore possible that the shift in firing patterns by cocaine exposure is caused by cocaine-induced alterations in conductance through these channels.

Increased input resistance and tonic discharge in PVT following cocaine may be the result of drug-induced changes in various neurotransmitters known to be expressed in the PVT. One neurotransmitter that may influence the excitability of PVT neurons following cocaine exposure is orexin. The PVT strongly expresses mRNA and protein for both orexin receptors 1 and 2 (Marcus et al., 2001), and also receives dense orexinergic innervation from the hypothalamus (Peyron et al., 1998). Subpopulations of neurons within the PVT are excited by both orexin A and orexin B (Bayer et al., 2002; Kolaj et al., 2007), and exposure to orexin neuropeptides have been shown to increase input resistance resulting from closure of K^+^ channels (Ishibashi et al., 2005). Interestingly, we recently showed that 7 days of cocaine exposure enhanced excitatory drive to PF/LHA orexin neurons (Yeoh et al., 2012). In addition to orexin, vasopressin has also been shown to cause state-dependent modulation of firing patterns in PVT neurons (Zhang et al., 2006). For example, exogenous application of arginine vasopressin produced membrane depolarization and tonic firing in 50% of the cells tested (Zhang et al., 2006). However, it remains to be determined how these neurotransmitters modulate the activity of thalamic neurons. Given that the PVT is a major target of these neurons, it is likely that their input to the PVT may be altered following cocaine exposure.

In addition to the potential for hypothalamic and vasopressin signalling to modulate the firing properties of PVT neurones, there is evidence of significant monamine inputs to this brain region (Hadfield and Milio, 1992; Otake and Ruggiero, 1995). To our knowledge, few electrophyphsiological studies have assessed the effects of dopamine on PVT neurons, however PVT neurons express dopamine D3 receptors and the D2/3 antagonist raclopride prevents psychostimulant-induced Fos-expression in these neurones (Deutch et al., 1998). Further to this point, noradrenaline is known to influence the conductance of PVT neurons. Specifically, noradrenaline can depolarize or hyperpolarize PVT neurons through actions on either postsynaptic α1 receptors or postsynaptic G-protein-coupled K^+^ channels α2 receptors (Heilbronner and Flügge, 2005). Finally, cocaine has also been shown to alter neuronal excitability of thalamic neurons by increasing serotonin levels within the thalamus (Hadfield and Milio, 1992). Together, this data suggests that further work investigating the potential role for monamines on the cocaine-induced effects we report here in PVT are warranted.

### Effects of CART on PVT neurons

The PVT was highlighted in key studies by Kelley et al. (2005) as a likely integrator of reward-seeking behavior. Significant work has now accumulated to strongly support this hypothesis (Hamlin et al., 2009; James et al., 2010, 2011b; Marchant et al., 2010; Martin-Fardon and Boutrel, 2012; James and Dayas, 2013). For example, cocaine dose-dependently increases Fos-protein expression in the PVT (Deutch et al., 1998), lesions of this region prevent cocaine-induced locomotor activity sensitization, (Young and Deutch, 1998) and conditioned place preference (Browning et al., 2014). Moreover, presentation of drug-associated cues and contexts also increases activation of PVT neurons (Brown et al., 1992; Franklin and Druhan, 2000; Dayas et al., 2008; James et al., 2011b) and inactivation of the PVT prevents reinstatement of drug-seeking (Hamlin et al., 2009; James et al., 2010).

While most addiction studies have focused on orexinergic inputs to the PVT, anatomical tracing work shows that CART terminals also innervate the entire rostro-caudal extent of the PVT (Kirouac et al., 2006). These projections arise from the arcuate nucleus, lateral hypothalamus, zona incerta and along the periventricular regions of the hypothalamus (Kirouac et al., 2006). Interestingly, CART-positive terminals are closely apposed to PVT neurons that project to the NAC shell (Parsons et al., 2006, 2007), and those that are responsive to drug cues (Dayas et al., 2008). Because CART has been shown to negatively regulate feeding behavior (Kristensen et al., 1998), we hypothesized that CART may have similar effects on drug-seeking and that these effects might be mediated by the PVT. Accordingly, we demonstrated that intra-PVT infusions of CART attenuated drug-primed reinstatement of cocaine-seeking (James et al., 2010). Consistent with these findings, the present study confirms that bath application of CART can significantly reduce sEPSCs frequency and amplitude in cocaine-exposed animals.

It is important to highlight that in the present study, recordings were made from aPVT neurons. CART fibers innervate both aPVT and pPVT to a similar extent and previously we showed that TTX and CART injections made across the anterior-posterior extent of the PVT were effective in reducing drug-primed reinstatement (James et al., 2010). However, recent studies have highlighted significant anatomical and functional differences between aPVT and pPVT divisions. For example, Flagel et al. (2011) showed that c-fos expression was selectively increased in the aPVT of animals that attributed incentive salience to food cues (Flagel et al., 2011). Furthermore, Li and Kirouac (2012) showed that the aPVT is more strongly innervated by the hippocampal subiculum and the prelimbic cortex, whereas the pPVT appears to receive inputs from the anterior most aspect of the prelimbic cortex and the agranular portions of the posterior insular cortex (see also Vertes and Hoover, 2008; Li and Kirouac, 2012). Together these studies highlight a future need to investigate potential rostro-caudal differences in cocaine-induced neuroadaptations to PVT neurons.

The exact signaling pathways through which CART regulates reward-seeking behavior in the PVT have yet to be determined, and indeed, the receptor for CART has yet to be identified. Whilst our experiment did not specifically determine the exact loci of CART actions, we showed that CART application reduced the sEPSC amplitude in PVT neurons, which is indicative of a postsynaptic mechanism. Importantly, many other studies have found that CART predominantly acts via postsynaptic mechanisms. For example, application of CART in hippocampal neurons in the presence of L-type Ca^2+^ channel agonist, Bay K 8664, caused a robust inhibition of depolarization-activated Ca^2+^ signals, which indicate that voltage-gated Ca^2+^ channels are targets for CART peptide, an effect which was blocked by pertussis toxin, suggesting the involvement of Gi/o proteins (Yermolaieva et al., 2001). Nevertheless, further studies will be required to determine the precise signaling pathways through which CART exerts its effects in PVT neurons.

Finally, we did not measure locomotor sensitization to cocaine injections, however a number of other studies have demonstrated that cocaine exposure as low as 10 mg/kg and up to 30mg/kg is able to induce locomotor sensitization in mice (Cunningham et al., 1999; Eisener-Dorman et al., 2011; Pascoli et al., 2012). It is reasonable to assume that repeated exposure to 15 mg/kg cocaine in the present study likely resulted in locomotor sensitization. Future studies will now be necessary to more closely examine whether changes in the excitability of PVT neurons is directly associated with the expression of sensitization.

## Conclusions

The present study has provided new insights into the effects of cocaine onto PVT neurons. We showed that cocaine exposure increases the proportion of PVT neurons that display a tonic firing AP discharge pattern. Further work will be required to understand what cellular and molecular changes within PVT neurons might drive this conversion, however, the net outcome of this cocaine-induced modification is a more excitable aPVT. Our study also provides clear evidence for an inhibitory effect of CART on excitatory input to PVT neurons and therefore extends our understanding of how this peptide might effects reward-seeking. In future studies, it will be important to assess the effect of CART on PVT neuron activity after extended cocaine consumption or multiple cycles of reward, to assess the relevance of this signaling to prolonged human addiction. Further work is also required to fully delineate the exact loci of CART’s actions in PVT, efforts that will benefit from the identification of CART receptors and the development of small molecular inhibitors.

## Conflict of interest statement

The authors declare that the research was conducted in the absence of any commercial or financial relationships that could be construed as a potential conflict of interest.
